# The effects of two Chinese herbal medicinal formulae vs. placebo controls for treatment of allergic rhinitis: a randomised controlled trial

**DOI:** 10.1186/1745-6215-15-261

**Published:** 2014-07-02

**Authors:** Rose YP Chan, Wai Tong Chien

**Affiliations:** 1 School of Nursing, Faculty of Health and Social Sciences, The Hong Kong Polytechnic University, Hung Hom, Kowloon, Hong Kong, SAR, China

**Keywords:** Allergic rhinitis, Body constitution, Chinese herbal medicine, Quality of life, Nursing students, Randomised controlled trial, Symptom severity

## Abstract

**Background:**

Allergic rhinitis is a chronic illness, affecting 10 to 40% of the worldwide population. Chinese herbal medicines, the treatment of allergic rhinitis, adopted thousands of years in ancient China, has recently raised much attention among researchers globally. This study evaluates the effects of two Chinese herbal formulae [Cure-allergic-rhinitis Syrup (CS) and Yu-ping-feng San (YS)] in treating undergraduate nursing students with allergic rhinitis over a 3-month follow-up, when compared to a placebo control group.

**Methods:**

A double-blind, randomised controlled trial with repeated-measures, three-parallel-groups design was conducted in a random sample of 249 participants recruited from one university in Hong Kong. After baseline measurements, participants were randomly assigned to CS, YS, or placebo groups (n = 83 per group). The main outcomes, including symptom severity, quality of life, and body constitution, were measured with self-administered questionnaires at baseline and immediately, 1 and 3 months after the 4-week interventions.

**Results:**

240 participants completed the trial, with 9 (3.6%) drop-outs. The results of Generalised Estimating Equations test followed by pairwise contrasts tests indicated that the participants who received CS showed significantly greater reduction of symptoms (mean difference of CS vs. placebo = 26.13–34.55, *P* <0.0005) and improvements in quality of life (mean difference of CS vs. placebo = 12.81–16.76, *P* <0.001), and body constitution in ‘Qi-deficiency’, ‘Yang-deficiency’, and ‘Inherited Special’ (mean difference of CS vs. placebo = 7.05–8.12, 7.56–8.92, and 4.48–8.10, *P* = 0.01– < 0.0005, 0.001–0.004, and 0.01– < 0.0005, accordingly, at three post-tests). The participants who received YS also indicated significant greater improvements in symptom severity, quality of life, and a few patterns of body constitution when compared to the placebo group. However, its effects were lesser in strength (i.e., smaller effect sizes), varieties of symptoms, and body constitution and sustainability over the 3 months.

**Conclusions:**

The herbal formula CS was found effective to reduce symptoms and enhance quality of life in young adults (nursing students) with allergic rhinitis in ‘Yang- and/or Qi-deficiency’ body constitution. Further controlled trials of its effects in Chinese and/or Asians with allergic rhinitis in terms of socio-demographic, ethnic and illness characteristics and a longer-term follow-up are recommended.

**Trial registration:**

The trial has registered at ClinicalTrials.gov with an ID: NCT02027194 (3 January 2014).

## Background

Allergic rhinitis (AR) is an allergic inflammation of the nasal airway induced by allergens such as dust, animal dander, or pollen. This illness imposes irritable symptoms such as nasal blockage, running nose, and repeated sneezing, and causes fatigue, sleep deprivation, and an inability to concentrate [1]. About 40% of the world population is affected by AR, which is expected to be more prevalent due to global warming and poor air quality [2,3]. Preventive measures, such as avoiding exposure to allergens, and pharmacological treatments, such as intranasal corticosteroids, can only be effective to control or reduce symptoms of AR, but are not able to cure the illness [4]. In addition, long-term medication use may result in a wide variety of adverse effects, ranging from mild dizziness, pain, and fatigue, to more serious headaches, palpitation, nose bleeding, and nasal polyps, and even hypertension and other cardiovascular problems [5]. As current treatments are not curative and treatment compliance is often not satisfactory [6], further research is essential to test an alternative treatment of AR with better curative or longer-term, and on the other hand less adverse, effects. Chinese herbal medicine (CM), which may produce possible curative function to a variety of chronic illnesses, has recently gained much interest and is increasingly tested and adopted for the treatment of AR.

Similar to Western medicine, in CM, the clinical manifestations (signs and symptoms) are collected from patients and interpreted for making diagnosis of a disease. However, CM practitioners diagnose a disease and prescribe treatment in terms of individual patterns of syndrome (‘*Zheng*’) and body constitution, reflecting an individual’s overall body condition [7]. Syndromes can be described as the pathological characteristics that an individual presents in response to the pathogens, allergens, or other undesirable stimulations; whereas body constitution is used to describe one’s overall physical and psychological condition [8]. Body constitution and current health status of an individual can be considered in terms of the balance between co-existing ‘Yin’ and ‘Yang’ (i.e., believed to be the physical form and functioning of a human being with low or deficient and high or excessive energetic qualities, respectively) and ‘Heat’ and ‘Cold’ conditions of his/her internal organs [8,9]. Therefore, ‘Yin’ (or ‘Cold’) in the body will become in excess when ‘Yang’ (or ‘Heat’) is lacking or deficient of energy, and vice versa. Treatments from the perspective of CM aim at helping an individual regain his/her balance between ‘Yin’ and ‘Yang’.

Based on these principles of CM, treatments for people with AR can be prescribed in consideration of their patterns of body constitution (or ‘*Zheng*’) [10]; most of whom have been identified as having two patterns of body constitution. These patterns include ‘Qi-deficiency’ (i.e., lack of energy, oxygen and blood to keep warm, and immune defence against allergens) during an early stage of AR [11] and ‘Yang-deficiency’ (i.e., unable to maintain normal function of the internal organs) in later (chronic) stage of the illness, particularly in their ‘Lung’ (respiratory), ‘Spleen’ (digestive), and ‘Kidney’ (excretory) organs [12]. Consequently, people with AR usually present with ‘cold’ or ‘flu’-like symptoms such as running nose, nasal congestion, and sneezing. As such, it is believed that an application of herbal medicine with ‘hot’ (‘Heat’) nature can strengthen functions of the ‘Lung’, ‘Spleen’, and/or ‘Kidney’ organs, thus alleviating the symptoms of AR and even curing the illness [13]. Nevertheless, a minority of patients with AR (<5%) can present with other patterns of body constitution, such as ‘Yin-deficiency’ with ‘Yang-hyperactivity’ [14], and thus require a different medicinal treatment from the majority of those with ‘Qi-deficiency’ and ‘Yang-deficiency’ as described above. Seven randomised controlled trials on the effects of Chinese herbal medicinal treatment of AR [15-21] were performed between 2000 and 2013 (Table 1). Different methods of assessment and evaluation on treatment outcomes (e.g., skin and multiple allergen tests, Ear, Nose and Throat specialty assessment, and blood samples for serum IgE, liver, and/or renal function tests) were used, mostly from the approaches to Western medicine. However, none of these trials adopted the ‘*Zheng*’ differentiation for prescription of treatment and, consequently, the prescribed Chinese herbal medicines varied greatly with regards to type and dosage which may not have been appropriate to the body constitution patterns presented by the patients receiving the medication. In addition, a very short-term follow-up of the treatment effects had been conducted, ranging from immediately to four weeks after completion of the treatment regimen. However, about half of these trials [16-18], the treatment group using Chinese herbal medicine reported non-significant improvements in most of their clinical outcomes, particularly with regards to symptom severity and health-related quality of life, when compared to the placebo control group.

**Table 1:** Seven randomised controlled trials of herbal formulae for people with allergic rhinitis (2000–2013)

**Study**	**Authors**	**Herbal formula**	**Baseline assessment**	**Sample and design**	**Treatment F/U**	**Main results**
Chinese Herbal Nasal Drop	Chui et al., (2010) [15]	Yu-ping-feng San:	Interviewed by Chinese medicine practitioner and Western physician	35 (20 and 15 in treatment and control group, respectively)	2 weeks treatment	Significant improvements in clinical symptoms: sneezing, itchiness, running nose, and stuffiness, as well as quality of life in terms of complexion and sleep
		*Radix Ledebouriellae* 5%			Follow-ups at the 2^nd^, 5^th^, and 7^th^ week of treatment (2 + 3 + 2 weeks) after intervention	
				14–71 years old		
		Others:		Two-groups, cross-over design		
		*Radix Glycyrrhizae* 6%	Outcomes:			
		*Herba Centipede* 23%	Clinical symptom score,			
		*Herba Menthae* 16%	Quality of life (ChQOL questionnaire)			
		*Radix Paeoniae Alba* 16%				
			Blood test for RFT, LFT, haematological status, and C-reactive protein			
		*Radix Scutellariae* 10%				
		*Radix Platcodi* 6%				
		*Floz Lonicerae* 5%				
		*Fructus Zizyphi Jujubae* 5%				
		*Rhizoma Coptidis* 4%				
		*Pericarpium Citri Reticulatae* 4%				
Shi-Bi-Lin	Zhao et al., (2009) [17]	Yu-ping-feng San:	ENT specialist assessment	126 (both 63 in treatment and control group)	4 weeks treatment (twice daily) and 5 visits	Significant improvements in symptom diary and severity, and quality of life
			Skin test for dust mite, mould, and animal dander		Baseline measurement and each week of intervention;	
		*Saposhnikovia divaricata* 7.5 g		18–65 years old		
				Double-blinded RCT, with placebo control		
			Blood test for LFT and full blood count			
			Outcomes:			Medical overall assessment not reached statistical significance
					2 weeks follow-up after intervention	
		*Magnolia biondii Pamp* 15 g	Blood serum IgE, Eosinophil Cationic Protein (ECP), symptom records, medicine diary, quality of life (SF-36), overall medical condition, and level of compliance			
						Blood test for IgE and ECP also not significant difference
		*Xanthium Sibiricum Patrin ex wider* 7.5 g				
		Others:				
		*Angelica dahurica* 20 g				
		*Gentiana scabra Bunge* 5 g				
		*Verbena officinalis* L. 5 g				
Bu-Zhong-Yi-qi-tang	Yang & Yu, (2008) [20]	*Formula ‘BZYQT*, included:	Skin test and MAST dust mite	60 (36 and 24 in treatment and control group, respectively)	3 months treatment (BZYQT & Ping-wai-san) in powder form; 3 times/day after meal	Nasal score, IgE level, PGE2 and LTC4 significantly decreased immediately after intervention
		Yu-ping-feng San:				
		*Atractylodes macrocephala Koidz* 2.0 g				
				17–32 years old	Pre- and post-test only	
				Perennial AR only		
			Outcomes:			
				RCT, comparing between two herbal formulae		
		*Astragalus mongholicus Bunge* 6.0 g	Nasal symptoms of Okuda and co-workers (1984); blood test for IgE, and measurement of E2 and LTC4, and COX smRNA			
		Others				
		*Panax ginseng C. A. Mey.* 4.0 g				
		*Citrus reticulate Blanco* 2.0 g				
		*Cimicifuga*				
		*foetida L.* 1.0 g *Angelica dah0rica fisch. ex Hoffim* 2.0 g				
		*Bupleurum chinense* DC 1.0 g				
		*Glycyrrhiza uralensis Fisch* 4.0 g				
		*Ziziphus jujube Mill var. inermis Rehd* 2.0 g				
		*Zingiber officinale Rosc* 3.0 g				
		Ping-wei-san:				
		*Atractylodes lancea Thunb* 4.0 g				
		*Magnolia officinalis Rehd. Et Wils* 1.5 g				
		*Citrus reticulate Blanco* 1.0 g				
		*Zingiber officinale Rosc* 0.5 g				
		*Ziziphus jujube Mill. var. inermis Rdhd* 0.5 g				
		*Glycyrrhiza uralensis Fisch* 1.0 g				
Xin-Yi-San	Yang et al., (2010) [21]	Yu-ping-feng San:	Skin test for dust mite, mould and animal dander	108 (62 and 38 in treatment and placebo control group, respectively)	3 months treatment (treatment and placebo groups) powders in capsule	Significant improvements in serum IgE level and nasal symptoms
			Outcomes:			
			Multiple allergen simultaneous test, nasal symptoms, nasal airflow resistance, nostril dissection area, and serum titer of IgE			
				18–64 years old RCT with placebo controls		
					Pre- and post-test only	
		*Saposhnikovia divaricata schischk*				
		Others:				
		*Rhizome og Glycyrrhiza uralensis Fisch*				
		*Magnolia Lilifloar desr*				
		*Asarum heterotropoides*				
		*Angelica dahu-rica Benth. Et Hook*				
		*Rhizome of Liqusticum sinense Oliva*				
		*Dried rhizomas of liqusticum Wallichi Franch*				
		*Rhizomas of Cimicifuga foetida L.*				
		*Rhizomas of Akebia quinata Decne*				
Biminne	Hu et al., (2002) [18]	Yu-ping-feng San:	Assessed by physician and skin test	58 (26 and 32 for treatment and placebo control group, respectively) 18–65 year old	Five capsules of herbal extract for 12 weeks and 5 visits	Non-significant improvement in symptom severity symptom improvements in treatment group and only sneezing was significantly improved
		*Ledebouriella divaricata* 460 g	Outcomes:			
		*Astragalus membranaceus* 552 g	Serum IgE, symptom diary with visual analogue scale, quality of life (RQLQ), patient evaluation of symptom improvement, and overall medical evaluation, and level of compliance	RCT with placebo controls	Baseline, interim and a few weeks after treatments	Quality of life was significantly improved
		Others:		One year later treatment was offered to the placebo (wait-list) controls		
		*Rehmannia glutinosa* 460 g				
		*Scutellaria baicalensis* 460 g				
		*Polygonatum sibiricum* 368 g				
		*Ginkgo biloba* 460 g				
		*Epimedium sagittatum* 460 g				
		*Psoralea corylifolia* 460 g				
		*Schisandra chinensis* 368 g				
		Pulp of *prunus mume* 184 g				
		*Angelica dahurica* 368 g				
Chinese herbal medicine	Xue et al., (2003) [19]	Yu-ping-feng San*:*	Assessed by Western (ENT) and Chinese medicine practitioners, conforming the diagnosis of AR	55 (28 and 27 for treatment and control group, respectively)	8 weeks of extract capsules	Significant improvements in symptom severity scores and quality of life in treatment group in the 8^th^ and 10^th^ week
		*Astragali, radix*				
					Pre- and post-test and 2 weeks after treatment	
		*Atractylodis macrofephalae rhizome*				
				27–54 years old		
		*Saposhnikoviae, radix*	Outcomes:	Subjects randomly allocated by computer		
		Others:	Nasal Symptom Quality of life (RQLQ), amount of medication use and blood tests for IgE, IgA, and IgM			
		*Xanthii, fructus*				
		*Angelicae Sinensis, radix*				
		*Asari, herba*				
		*Bupleuri, radix*				
		*Cimicifugae, rhizome*				
		*Codonopsis pilosulae, radix*				
		*Chuanxiong, rhizome*				
		*Magnolia, flos*				
		*Menthae, herba*				
		*Citri reticulatae, pericappium*				
		*Plantaginis, semen*				
		*Schisandrae, fructus*				
		*Schizonepetae, herba*				
		*Chebulae, fructus*				
RCM-102	Lenon et al., 2012 [16]	Yu-ping-feng San:	Medical assessment by ENT expert	95 (47/48)	8 weeks of treatment – two capsules 3 times per day (treatment and placebo group)	No significant differences on all outcomes
				18–65 years old		
		*Astragalus membranaceus* (Fish.)				
			Outcomes:			
			Skin test, symptom severity, quality of life, and blood test for liver and kidney functions			
		*Saposhnikovia divaricate (Turcz).*				
					Bi-weekly F/U till the end of treatment	
		*Glycyrrhiza uralensis* (Fisch.)				
		Others:				
		*Bupleurum Chinense D.C.*				
		*Magnolia Liliflora (Desr.)*				
		*Mentha haplocalyx Briq.*				
		*Schizonepeat Tenuifolia Briq*				
		*Scutellaria baicalensis Geogi.*				

Yu-ping-feng San (YS), or its modified form, is one of the common herbal medicinal formulae with a ‘hot’ (‘Heat’) nature, and is recommended for enhancing the functions of the ‘Lung’ and ‘Spleen’ organs when treating several types of respiratory diseases such as AR [22-24]. Two recent CM studies reported that people with AR who received a 4-week YS treatment reported a significant reduction of AR symptoms and serum IgE or allergen reactivity at immediately and/or 2–4 weeks after intervention [22,24]. Similar to the abovementioned controlled trials [15-21], the YS could not consistently demonstrate significant therapeutic effects on most of the other clinical outcomes such as patients’ body constitution and quality of life, as well as its longer-term effects (e.g., >8 weeks).

Another herbal medicinal formula termed Cure-allergic-rhinitis Syrup (CS), which consists of three classical herbal formula ‘Huang qi jian zhong tang’, ‘Li zhong tang’, and ‘Gui zhi tang’, has been used for over thousands of years for the treatment of people with ‘Cold’ and ‘Qi-deficiency’ body constitution and weaknesses in ‘Lung’ and ‘Spleen’ organs, as well as in ‘Kidney’ [25], particularly those with respiratory illnesses such as AR [26-28]. The CS is also ‘hot’ in nature and has been suggested to enhance the functions of the ‘Lung’ and ‘Spleen’ organs, but also the ‘Kidney’, which all together are believed to be the main reasons (‘roots’) of AR [27,28]. Therefore, people who have been diagnosed with a ‘Qi-deficiency’ and/or ‘Yang-deficiency’ body constitution (or *‘Zheng’*) are considered appropriate and beneficial to receive the CS treatment.

This study aimed to evaluate and compare the effectiveness between two alternative herbal medicines, CS and YS, and one placebo group for young adults (nursing students) with AR on improving their AR symptoms (primary outcome), three ‘unhealthy’ patterns of body constitution (‘Qi-deficiency’, ‘Yang-deficiency’, and ‘Inherited special’), and health-related quality of life, over a 3-month follow-up. This double-blind controlled trial was the first one in Chinese young adults with AR to test the effectiveness of the two Chinese herbal medicinal formulae on patient outcomes from the perspectives of both Chinese and Western medicine (e.g., body constitution and AR symptoms, respectively). The research hypothesis for the primary outcome was that the participants in the CS group and/or the YS group would indicate a significant reduction of their AR symptom severity than those in the placebo group immediately and at 1 and 3 months after completion of their 4-week treatments. Secondary hypotheses included that the CS and/or the YS participants would indicate significant improvements in their health-related quality of life and/or body constitution than those in the placebo group over the 3-month follow-up. We would also like to test whether the treatment effects of CS would be significantly greater than those of YS or not.

## Methods

This was a double-blind randomized controlled trial with repeated-measures, three parallel groups (i.e., two comparative treatment groups and one placebo control group) design. A random sample of nursing students considered one of the highly prevalent groups of AR was recruited as subjects in this controlled trial. The nursing students with AR who were assessed and found eligible for inclusion (n = 249) and completed the baseline measures (T1) were randomly assigned into one of the three study arms, including the Cure-allergic-rhinitis syrup (CS), Yu-ping-feng San (YS), or placebo control group, each consisting of 83 participants.

**Figure 1:**
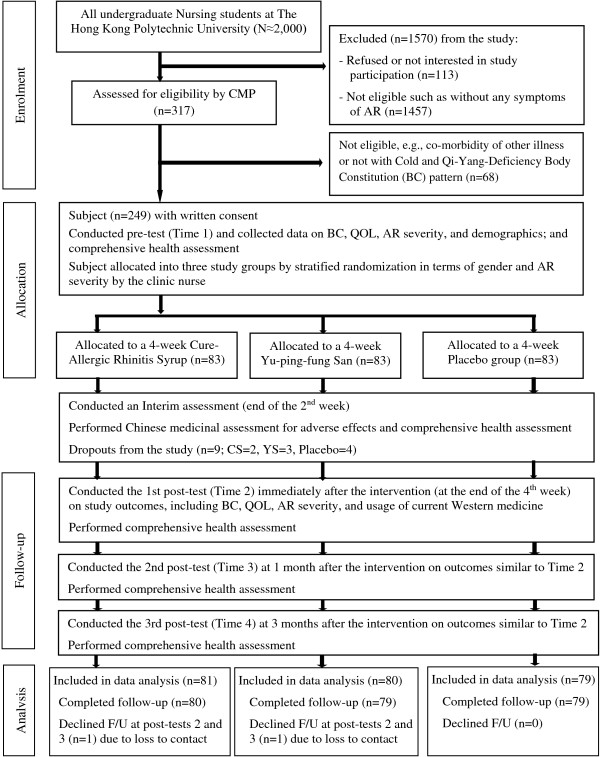
**A flow diagram of the procedure of this clinical trial.** After confirming eligibility, 249 nursing students were recruited and randomly assigned into one of the three study arms after completing the baseline measurements; 240 completed one to three post-tests over a 3-month follow-up and finally only 9 dropped out during the treatment period.

Sample recruitment, medical assessments, prescriptions, and outcome measurements and follow-ups were performed at the Integrative Health Clinic of the Hong Kong Polytechnic University serving for students, staff, and visitors between April 2013 and February 2014. Figure 1 shows the flow diagram of the controlled trial procedure according to the revised version of the CONSORT statement [29], including subject enrolment, group allocation, outcome measurements, follow-ups, and data analyses.

### Recruitment

Participants were randomly recruited from the baccalaureate degree nursing programmes at one of the three universities with similar full-time undergraduate nursing programmes (about 35% of the total nursing students) in Hong Kong. There were about 2,000 full-time undergraduate nursing students at the Hong Kong Polytechnic University under study during recruitment. When attending classes, the students who agreed to use CM and participate in this study (n = 319) were asked to attend medical assessment by a registered CM practitioner in the Integrative Health Clinic. Those who met the study criteria specified below (n = 249) were asked to complete a written consent with full explanation of the study procedure and were then grouped in terms of gender and AR symptom severity (mild, moderate, and severe). For each of the six groups of eligible subjects, each student was asked to draw a labelled card (1 = CS, 2 = YS, and 3 = Placebo) from an opaque envelope and was then allocated into the CS, YS, or placebo group for the 4-week treatment.

Inclusion criteria of participants specified those full-time undergraduate nursing students who were: (a) previously diagnosed with AR (seasonal or perennial) by a Western or CM practitioner for >1 year with at least one allergic history; (b) presenting a body constitution of ‘Qi-deficiency’ and/or ‘Yang-deficiency’, according to the Chinese medicinal assessment at recruitment; (c) aged 18 or above; and (d) able to understand Cantonese or Mandarin and the written Chinese language in the self-reporting questionnaires. Exclusion criteria for the nursing students included those who: (a) presented a body constitution of ‘Heat’ and/or ‘Yin-deficiency’ during the CM assessment; (b) were currently receiving Chinese and/or Western medications such as anti-hypertensive and psychiatric drugs; (c) had any known medical history or co-morbidity of one or more acute or chronic medical diseases, such as heart, liver, and lung diseases, and mental disorders; (d) had recently received a major operation, chemotherapy and/or radiotherapy, or planned to be hospitalised over the study period; and (e) had any known history of allergy to Chinese herbal medicines.

### Sample size calculation

Sample size calculation was based on the average effect sizes in two previous clinical trials of Chinese herbal medicine for people with AR [21,30] on improving the severity of AR symptoms (primary outcome), of 0.36 (ranging from 0.30 to 0.44), at one week after treatment, when compared to the placebo group. With the level of statistical significance set at 0.05 and a study power of 90% [31], an estimated sample size was 63 participants per group (i.e., 226 nursing students in three study arms) using the formula of Cohen’s *d* for repeated-measures ANOVA test among three groups in *GPower* software [32], taking account of a 20% potential attrition [21]. Hence, all of the students (n = 249) who met the study criteria and agreed to participate were included because they were similar to the estimated sample size calculated for achieving a satisfactory power and statistical significance in this study.

### Allocation and randomization

A stratified random sampling method from three groups of eligible subjects in terms of symptom severity of AR (mild, moderate, and severe) was adopted according to the assessment and survey guideline of the World Health Organization [33]. Each symptom severity group was further subdivided into two subgroups in terms of gender before drawing a labelled card for group allocation. These two characteristics were the potential confounding factors of treatment effects in AR from the perspective of CM [34]. The medical assessment, randomisation procedure, and treatment allocation were performed by an independent clinic nurse, while the participants, assessor (trained research assistant), and researchers were fully concealed and blinded to the group assignment and treatment procedure. In order to maintain complete blinding, a clinic nurse safely kept the participant list locked and, with assistance from clinic staff, administered bottles of Chinese herbal medicine according to the treatment (study group) assignment.

### Interventions

Two types of herbal medicine were prepared and bottled by the researcher and kept in the refrigerator of the clinic one day before use. The herbal and placebo medicines were in syrup form due to better taste and absorption and user-friendliness. A very small amount of fresh ginger (3 g) was added to the placebo medication to establish a spicy taste and smell and be able to exert seasoning but no treatment effect in AR [18,30]. With sugar and wheat powder added to the herbal medicines, the spicy taste and texture of the three kinds of syrups used for all of the three study groups were very similar. This similar form, colour, and route of administration of the medication used among the three study groups was beneficial to provide a consistent and standardized format of medicinal treatment in this trial, as well as high levels of convenience and preference for people with chronic AR disease. The same decoction method for the CS, YS, and placebo medication included: (a) after the cleansing procedure, boiling the herbal medicine with 2,000 mL of water for 2 hours to 500 mL of herbal concoction; (b) after cooling to room temperature, pouring the 500 mL of herbal concoction into a glass bottle (750 mL or 1,000 mL); and (c) labelling it as 1 (=CS), 2 (=YS), or 3 (=placebo) and storing it in a refrigerator (at 4°C). Each of the participants in the three study groups was provided with one bottle of 500 mL herbal syrup weekly for four weeks after a Chinese medicinal consultation and assessment made in the clinic. A standard spoon was offered to each participant to administer 70 mL of syrup once per day from the bottle to be diluted with half a cup of water. Participants could easily transport the diluted syrup bottle to any school, extracurricular, or social activity at their own convenience.

The Cure-allergic-rhinitis Syrup [治敏膏] (CS) contained 12 herbal medicines with a ‘hot’ nature, based on three classical herbal formulas – ‘Huang qi jian zhong tang’, ‘Li zhong tang’, and ‘Gui zhi tang’ [25]; its ingredients with their respective functions are outlined in Table 2. The composition and dosage of the CS were reviewed and approved by a group of experienced CM practitioners and pilot tested in 20 young adults with AR by the researchers with satisfactory therapeutic effects on symptom reduction and no adverse effect noted during interim and post-intervention assessments by two CM practitioners. The CS had to be taken once daily (70 mL) over 4 weeks in this study.

**Table 2:** Content and nature of 12 Chinese herbs in Cure-allergic-rhinitis Syrup (CS)

**Pharmaceutical name**	**Chinese name**	**Nature**	**Flavour**	**Acting organs**	**Functions**
*Astragali Radix* (6 g)	黃耆 Hyangqi	Slight warm	Sweet	Lung, spleen	Reinforce Qi and strengthen body resistance
*Codonopsitis Radix* (6 g)	黨参 Dan Shen	Neutral	Sweet	Lung, spleen	Tonify the lung and spleen
*Atractylodis macrocephalae Rhizoma* (6 g)	白朮 Baizhu	Warm	Sweet bitter	Spleen	Reinforce Qi of the spleen
*Rhizoma Zingiberis* (3 g)	乾薑 Ganjiang	Warm	Acrid bitter	Spleen, liver	Re-warm the spleen
*Cinnamomi Ramulus* (3 g)	桂枝 Guizhi	Warm	Acrid sweet	Lung, heart	Reinforce Qi and disperse cold
*Jujubae Fructus* (12 g)	大棗 Dazap	Warm	Sweet	Spleen	Reinforce Qi and digestive system and harmonise different drugs
*Aconitilaterali Radix sPreparata* (1.5 g)	熟附子 Fuzi	Hot	Acrid	Spleen, kidney	Reinforce Yang, expel ‘cold’ and release ‘flu’ syndrome
*Asari* Radix et Rhizoma (0.6 g)	細辛 Xixin	Warm	Acrid	Lung, kidney	Expel wind and cold, warm the lung, and decrease nasal congestion
*Magnoliae Flos* (3 g)	辛夷 Xinyi	Warm	Acrid	Lung	Expel ‘cold’ and decrease nasal congestion
*Folium Artemisiae* (3 g)	艾葉 Aiye	Warm	Bitter acrid	Spleen, kidney	Warm the meridians and dispel ‘cold’
*Triticum aestivum* (3 g)	淮小麥	Neutral	Sweat	Heart, liver	Soothe the liver and replenish Yin
*Saccharum Granorum*	飴糖 Yitang	Warm	Sweet	Lung, spleen	Strengthen and replenish the Qi of the spleen

A modified form of Yu-ping-feng San [玉屏風散] (YS), which contained six herbs previously used for treatment of AR in recent CM studies [22-24], was adopted to be an alternative medicinal treatment in this study. Similar to CS, the majority of the contents of this herbal medication were also ‘hot’ in nature; its ingredients and their functions are outlined in Table 3. Finally, the syrup for the placebo control group contained wheat powder, sugar, and a small amount of spicy-tasting fresh ginger, thus having a similar taste and smell as the above two herbal medicines used.

**Table 3:** Content and nature of six Chinese herbs in Yuk-ping-feng San (YS)

**Pharmaceutical name**	**Chinese name**	**Nature**	**Flavour**	**Acting organs**	**Functions**
*Astragali Radix* (15 g)	黃耆 Hyangqi	Slight warm	Sweet	Lung, spleen	Reinforce Qi and strengthen external body resistance
*Atractylodis macrocephalae Rhizome* (12 g)	白朮 baizhu	Warm	Sweet bitter	Spleen	Reinforce Qi of the spleen
*Saposhnikoviae Radix* (3 g)	防風 Fangfeng	Slight warm	Acrid	Spleen, liver	Expel wind-dampness and alleviate pain
*Magnolia Flos* (3 g)	辛夷 Xinyi	Warm	Acrid	Lung, stomach	Expel cold and decrease nasal congestion
*Glycyrrhiza uralensis*	甘草	Neutral	Sweet	Spleen, lung	Reinforce Qi and harmonise all other herbs
*Xanthii, fructus*	蒼耳子	warm	Acrid bitter	Lung	Release nose blockage and expel wind

Progress of the participants in each treatment and monitoring of their health condition was assured by interim medical assessment by the same CM practitioner by an appointment made at the individual’s convenience. Problems with the progress of medication taken and missed doses during the 4-week treatment were collected by the clinic nurse and discussed weekly among the research team and CM practitioner. Strategies in improving medication compliance and referrals to medical consultation for any reported adverse effects were encouraged and clarified with individual students in need of support. The participants also agreed not to inform the assessor and CM practitioner at the clinic about their study participation. Any queries on the study and their treatment used would be first discussed with the clinic nurse as needed.

### Measures

Participants completed three outcome measures within the questionnaires delivered by the research assistant and returned to her at recruitment (T1), immediately (T2), and at 1 (T3) and 3 months after completion of their 4-week treatment. These measures included patterns of body constitution with the Constitution in Chinese Medicine Questionnaire (CCMQ), symptom severity with the 10-cm visual analogue scales of the Rhinoconjunctivitis Quality of Life Questionnaire Standard (RQLQ-S), and quality of life with the 7-point Likert scales rated on items of the RQLQ-S.

#### ***Constitution in Chinese Medicine Questionnaire (CCMQ)***

The 60-item CCMQ was developed by Wang et al. [35] for evaluation of the patterns of body constitution and consisted of nine patterns, including eight unhealthy patterns, namely ‘Qi-deficiency’ (8 items), ‘Yang-deficiency’ (7 items), ‘Yin-deficiency’ (8 items), ‘Phlegm-dampness’ (8 items), ‘Dampness-heat’ (6 items), ‘Blood stasis’ (7 items), ‘Qi-depression’ (7 items), and ‘Inherited special’ (7 items), and one healthy ‘Gentleness’ (8 items). The nine patterns of body constitution have been commonly employed in assessment of patients with a variety of allergic reactions [36]. The participants rated their level of experienced symptoms over the past 7 days on a 5-point Likert scale, ranging from ‘1 = No’ to ‘5 = All of the time’. The total score of each pattern was calculated by adding up the scores of all items and subtracting the total number of items in the pattern, then divided by to the total number of items multiplied with four, and finally transforming into a percentage [35,37]. A higher percentage of the transformed score in the eight unhealthy patterns indicated a poorer health state, whereas a higher percentage in the ‘Gentleness’ represented a better overall health state. As the therapeutic effects of the CM treatment used are mainly aimed at helping the participants with ‘Qi-deficiency’, ‘Yang-deficiency’, and ‘Inherited special’, the outcome evaluation was focused on these three unhealthy patterns of body constitution in this study, as well as the ‘Gentleness’ indicating an overall healthy state of the participants.

The CCMQ has demonstrated satisfactory face validity and content validity (content validity index was 0.8) with a group of CM experts [35]. In addition, the overall scale and its subscales showed satisfactory concurrent validity with SF-12 (Pearson’s *r* = 0.18–0.61, *P* <0.01), reproducibility (kappa statistics ranged from 0.32–0.53), sensitivity in predicting patterns of body constitution and CM diagnoses (50%–75%), and internal consistency (Cronbach’s alpha coefficients of 0.71–0.88), in 1,084 Hong Kong Chinese people [38].

#### ***Rhinoconjunctivitis quality of life questionnaire standard (RQLQ-S) – visual analogue scales measuring symptom severity***

The 28-item RQLQ-S was modified from the original RQLQ developed by Juniper and Guyatt [39] with further testing on its validity in terms of symptom assessment and standardization of the first three questions. It consisted of seven domains, including symptoms in sleep (3 items), nasal (4 items), non-nasal/eye (7 items), practical problems (3 items), activity limitation (3 items), eye symptoms (4 items), and emotional function (4 items). Severity of symptoms of AR was assessed by self-rating on a set of 10-cm visual analogue scales, from a starting point denoted ‘No symptom’ to a 10 cm end-point denoted ‘Extremely severe’. The RQLQ-S was sensitive to detect a mild change in self-perceived symptom severity of AR; a change of 0.5 in mean total score of the RQLQ-S has been considered a clinical significant change in one’s disease condition of AR [40]. The RQLQ-S could also be applied to assess different levels of symptoms of AR, including mild to severe intermittent and persistent ones [41,42].

#### ***Rhinoconjunctivitis quality of life questionnaire standard (RQLQ-S) – 7-point Likert scales measuring quality of life***

The 28-item RQLQ-S was developed for evaluating the health-related quality of life of people with AR [43]. As mentioned above, in order to measure symptom severity the respondents indicated their perceptions toward their functional impairments in relation to AR over the past 7 days by rating on a 7-point Likert scale, ranging from ‘0 = None’ to ‘6 = Extremely’. With equal weighting on each item, the total scores for both the total scale and its seven subscales would be summed up from all of their item scores [40]. A higher total score would indicate a lower level of perceived quality of life. The scale has demonstrated a very strong correlation (Pearson’s r = 0.86) with the original RQLQ [39], and very satisfactory test-retest reliability (Intra-class correlation of 0.99, *P* = 0.001) and internal consistency (Cronbach’s alpha of 0.92) in 100 people with AR [43]. Its concurrent validity with physical and mental domains of SF-36 was also satisfactory (Pearson’s r = -0.35 and -0.47, respectively).

#### ***Demographic data sheet***

Socio-demographic data (e.g., age, gender, education, marital status, and family household income) and medical history (e.g., duration of AR, current medical treatment received and other co-morbidities) of the participants were collected by using a demographic data sheet designed by the researchers.

### Data collection procedure

The potential subjects (nursing students) who showed interest in this trial were assessed for their eligibility of participation by a registered CM practitioner in the Integrative Health Clinic of the University. For those eligible to participate, they were approached by the research assistant to explain the purpose and procedures of the study and obtain the written consent. They were then administered with three self-reporting questionnaires (i.e., the CCMQ, the RQLQ-S with visual analogue scales, and the RQLQ-S with 7-point Likert scales) and the demographic data sheet as the baseline measurement (T1). After completion of the questionnaires and being randomly allocated to either one of the three groups by drawing a labelled card, the clinic nurse administered a bottle of herbal medicine to the participants according to their assigned treatment. Participants returned to the clinic once a week to collect a new bottle of herbal medicine. At the second week, they received an interim medical assessment by the registered CM practitioner to monitor their treatment progress and detect any adverse effects experienced.

After the 4^th^ week of treatment, they returned to the clinic thrice for follow-up assessment on the three treatment outcomes (body constitution, symptom severity, and quality of life) by completing the same set of questionnaires as T1, at immediately (T2, by the end of the 4^th^ week), 1 month (T3), and 3 months (T4) after completion of their treatments. The participants were given between 20 and 25 minutes to complete the questionnaires. During each outcome measurement and interim assessment, they also received a comprehensive health assessment by the researchers, including their vital signs and oxygen saturation, peak flow rates to detect respiratory functioning and possible adverse effects of the treatment received.

### Ethical considerations

Ethical approval of this trial was obtained from the Human Subjects Research Ethics Committee of the Hong Kong Polytechnic University (Ref. No.: HSEARS20130111002). Written informed consent was obtained from the respondents after the study purpose and procedure had been fully explained. If any illness and compliance problems were found in any of these participants over the study period, they were assessed by the CM practitioner and referred to further medical advice if deemed necessary. Students were reminded of reporting any discomfort and co-morbid illness, if any, and encouraged to ask any questions about the study. They were given the emergency contact telephone of the clinic nurse and a researcher and were referred to Western and Chinese medical consultation as needed.

The herbal medicine selection was double-checked by an independent registered CM practitioner and medicinal formation was agreed by at least another two medical practitioners. Any toxic effect of the herbal medicines used was minimised by the optimal boiling time and low dosage. Although there would be very low risk for adverse events (<0.01%) [22], comprehensive health assessments were provided at baseline, interim assessment, and three post-tests for each of the participants to monitor their health parameters such as vital signs, body weight, and urine for routine test to detect any possible adverse effects of the herbal medicines used.

### Data analysis

Pre- and post-test data collected from the two treatment groups and the placebo group were numerically coded and analysed using the IBM SPSS for Windows, version 20. Goodness-of-fit *χ*^2^ test was used to test any differences in the demographic characteristics between the three groups. Mean score differences on the outcome measures at baseline were compared between the three groups using one-way analysis of variance test. Final data analysis based on intention-to-treat principle was performed using the Generalized Estimating Equation (GEE), which has been commonly used for comparisons among both categorical and continuous variables with or without normal distribution of scores [44], to examine and compare the mean scores of symptom severity and quality of life (two sets of ratings of RQLQ-S) and percentages of four patterns of body constitution (CCMQ) within and between three groups and across four time measurements (T1 to T4). Post-hoc (pair-wise) comparisons were then performed to test any significant differences on the mean scores of each outcome measure between the three groups at each of the three post-tests (T2 to T4) if the overall treatment effect was found significant in the GEE test. The level of significance of all statistical tests was set at 5%.

## Results

### Characteristics of participants at baseline

A total of 249 nursing students with AR were recruited and nine of them (3.6%) were unable to complete the 4-week treatment and were lost to follow-up. Therefore, 240 participants were finally included in the data analysis (Figure 1). The main reasons for withdrawal were too busy to follow the treatment regimen (n = 2, 0.8%), parental objection (n = 3, 1.2%), and experience of discomfort or mild adverse effects such as increased acne and abdominal distension (n = 4, 1.6%). Ten participants were unable to complete their treatment but were willing to be followed-up at post-tests and were therefore included in the data analysis (based on the intention-to-treat principle).

As indicated in Table 4, the majority of the 240 participants in the three groups was female (n = 146, 60.8%) and in Years 1 to 3 (n = 219, 91.3%) of their 4-year undergraduate nursing programmes. Their mean age was 21.30 years (SD = 1.64), which ranged from 18–25 years. Onset of AR in childhood was most common and more than half (n = 149, 62.1%) had AR in primary school or below. Two-thirds of them experienced attacks of irritable symptoms of AR from daily (n = 68, 28.3%) to several times per week (n = 66, 27.5%). More than half did not receive any kind of treatment for AR (n = 131, 54.6%), while 29 (12.1%) received either Chinese medicine only or both Chinese and Western medicine.

**Table 4:** Demographic and clinical characteristics of participants in the three study groups (n = 240)

**Characteristics**	**CS (n = 81)**		**YS (n = 80)**		**Placebo (n = 79)**		***χ***^**2**^	***P***
	**n**	**%**	**n**	**%**	**n**	**%**		
**Gender**							2.06	0.36
Male	27	33.33	32	40.00	35	44.30		
Female	54	66.67	48	60.00	44	55.70		
**Age (years)**							1.97	0.37
18–22	63	77.78	69	86.25	65	82.28		
23–25	18	22.22	11	13.75	14	17.72		
**Marriage**							1.03	0.60
Single	81	100.00	79	98.75	78	98.73		
Married	0	0.00	1	1.25	1	1.27		
**Family income (HK$)**^**a**^							5.24	0.87
Below 10,000	8	9.88	13	16.25	10	12.66		
10,000–19,999	23	28.39	29	36.25	27	34.18		
20,000–29,999	23	28.39	22	27.50	23	29.11		
30,000–39,000	17	20.99	10	12.50	15	18.99		
40,000 or above	10	12.35	3	3.75	4	5.06		
**Year of study**							7.37	0.29
Year 1	25	30.86	32	40.00	21	26.58		
Year 2	27	33.33	30	37.50	32	40.51		
Year 3	19	23.46	12	15.00	21	26.58		
Year 4	10	12.34	6	7.50	5	6.33		
**Onset of AR**							11.92	0.29
Before primary school	23	28.40	20	25.00	24	30.38		
Primary school	24	29.63	33	41.25	25	31.65		
Secondary school	31	38.27	20	25.00	26	32.91		
University	3	3.70	7	8.75	4	5.06		
**Frequency of AR attack**							15.36	0.12
Daily	24	29.63	18	22.50	26	32.91		
One to a few weekly	28	34.57	17	21.25	21	26.58		
Once to a few monthly	11	13.58	18	22.50	15	18.99		
Quarterly	18	22.22	27	33.75	17	21.52		
**Reasons for AR**							17.13	0.51
Allergens	24	29.63	32	40.00	27	34.18		
Allergens and cold temperature	57	70.37	48	60.00	52	65.82		
**History of Medical Treatments used**							6.43	0.60
None	42	51.85	48	60.00	41	51.90		
Western medicine and/or operation	29	35.80	24	30.00	27	34.18		
Chinese medicine	2	2.47	4	5.00	1	12.66		
Both Western and Chinese medicine	8	9.88	4	5.00	10	12.66		
**Family History of AR**							14.53	0.41
None	27	33.33	34	42.50	31	39.24		
Parent	18	22.22	15	18.75	10	12.66		
Sibling	25	30.87	18	22.50	20	25.32		
Parents and siblings	11	13.58	13	16.25	18	22.78		
**Other allergies**							10.68	0.71
None	44	55.32	47	58.75	48	60.76		
Skin allergy	23	28.40	21	26.25	22	27.85		
Food allergy	14	17.28	12	15.00	9	11.39		

CS, Cure-allergic-rhinitis Syrup group; YS, Yu-ping-feng San group; AR, Allergic rhinitis.

^a^US$1 = HK$7.8.

Further, more than half of the participants had a family history of AR (n = 148, 61.7%). The AR-induced allergens reported by the participants mainly included dust/polluted air, smoke, hair, mould, and chemicals (e.g., disinfectants), and pollen contact was reported by 18 participants only (7.5%). Less than half of the participants (n = 101, 42.1%) also had other allergies such as food allergies.

### Homogeneity of the sample in three study groups at baseline

An assessment of homogeneity of the characteristics and mean scores of the outcome measures of the three study groups was performed. As the results indicated in Table 4, there were no significant differences on the socio-demographic and clinical characteristics of the participants between the three study groups (*P* values ranged from 0.12 to 0.87). Mean scores (and standard deviations) of the outcome measures (CCMQ and RQLQ-S for both symptom severity and quality of life) at baseline measurement are summarised in Table 5. There were also no significant differences on the mean scores of the three outcome measures between the three study groups (*P* values ranged from 0.15 to 0.99), using one-way analysis of variance test.

**Table 5:** Comparison of mean scores of symptom severity, quality of life and body constitution at baseline between three groups (n = 240)

	**CS (n = 81)**		**YS (n = 80)**		**Placebo (n = 79)**		**ANOVA**	
**Variables**	**Mean**	**SD**	**Mean**	**SD**	**Mean**	**SD**	**F**	***P***
**RQLQ-S – Symptom severity**	102.93	45.04	101.59	49.35	105.93	46.84	0.18	0.84
**CCMQ**								
Gentleness	55.31	12.25	53.47	12.02	52.42	12.98	0.64	0.53
Qi-deficiency	42.85	15.95	39.56	16.93	44.83	15.24	1.92	0.15
Yang-deficiency	38.25	20.98	39.36	20.48	42.32	18.16	0.74	0.48
Inherited special	41.07	15.09	39.77	14.93	39.86	17.15	0.02	0.99
**RQLQ-S – Quality of life**	59.73	23.86	58.91	26.85	60.87	23.70	0.13	0.88

Outcome measures at baseline were compared between groups by using the one-way analysis of variance (ANOVA) test.

CS, Cure-allergic rhinitis Syrup group; YS, Yu-ping-feng San syrup group; RQLQ-S, Rhinoconjunctivitis Quality of Life Questionnaire Standard; CCMQ, Constitution in Chinese Medicine Questionnaire.

### Main treatment effects

Results of the interaction (Group × Time) effects using GEE (Table 6) indicated that there were statistically significant differences between the three study groups over the 3-month follow-up on symptom severity (Wald *χ*^2^ = 24.49, *P* <0.0005; effect size = 0.75), quality of life (Wald *χ*^2^ = 19.47, *P* = 0.003; effect size = 0.64), and two patterns of body constitution, including ‘Qi-deficiency’ (Wald *χ*^2^ = 15.98, *P* = 0.01; effect size = 0.40) and ‘Inherited special’ (Wald *χ*^2^ = 16.58, *P* = 0.01; effect size = 0.52). Although there were no significant interaction effects on the other two patterns of body constitution (‘Gentleness’ and ‘Yang-deficiency’), the mean scores in all of the three groups showed consistent improvements over time (i.e., within-group effects were statistically significant, Wald *χ*^2^ = 99.90 and 137.52 for ‘Gentleness’ and ‘Yang-deficiency’, respectively, and both *P* values <0.0005).

**Table 6:** Results on symptom severity, quality of life, and body constitution in the three groups at four measurements and comparisons using GEE (n = 240)

	**CS (n = 81)**				**YS (n = 80)**				**Placebo (n = 79)**				**GEE (Group × Time)**	
	**T1**	**T2**	**T3**	**T4**	**T1**	**T2**	**T3**	**T4**	**T1**	**T2**	**T3**	**T4**		
	**Mean**	**Mean**	**Mean**	**Mean**	**Mean**	**Mean**	**Mean**	**Mean**	**Mean**	**Mean**	**Mean**	**Mean**		
	**(SE)**	**(SE)**	**(SE)**	**(SE)**	**(SE)**	**(SE)**	**(SE)**	**(SE)**	**(SE)**	**(SE)**	**(SE)**	**(SE)**	**Wald *χ***^**2**^	***P***
**SS total score**	102.93 (5.33)	61.21 (5.33)	51.42 (5.33)	42.29 (5.33)	101.59 (5.37)	66.49 (5.37)	62.21 (5.37)	49.76 (5.37)	105.93 (5.40)	87.34 (5.40)	84.06 (5.40)	76.83 (5.40)	24.49	<0.0005
**QOL total score**	59.73 (2.78)	37.54 (2.78)	32.56 (2.78)	29.22 (2.78)	58.91 (2.80)	41.78 (2.80)	37.86 (2.80)	33.34 (2.80)	60.87 (2.82)	50.35 (2.82)	49.32 (2.82)	45.08 (2.82)	19.47	0.003
**Body constitution**														
Gentleness	55.31 (1.40)	60.90 (1.40)	61.99 (1.40)	61.91 (1.40)	53.47 (1.40)	57.73 (1.42)	59.15 (1.41)	60.64 (1.41)	52.42 (1.41)	54.72 (1.42)	56.06 (1.42)	59.97 (1.42)	9.02	0.17
Qi-deficiency	42.85 (1.80)	35.05 (1.80)	33.58 (1.80)	33.57 (1.80)	39.56 (1.80)	37.50 (1.82)	36.43 (1.82)	34.82 (1.82)	44.83 (1.81)	42.92 (1.83)	40.05 (1.83)	39.27 (1.82)	15.98	0.01
Yang-deficiency	38.25 (2.13)	29.46 (2.17)	28.57 (2.17)	28.26 (2.15)	39.36 (2.16)	34.54 (2.19)	33.96 (2.19)	33.29 (2.16)	42.32 (2.16)	37.99 (2.18)	34.99 (2.19)	35.17 (2.17)	6.75	0.35
Inherited special	41.07 (1.71)	32.43 (1.72)	31.33 (1.71)	29.36 (1.71)	39.77 (1.72)	34.37 (1.74)	32.13 (1.73)	32.09 (1.73)	39.86 (1.73)	37.65 (1.74)	33.91 (1.74)	36.49 (1.74)	16.58	0.01

SS, Symptom severity; QOL, Quality of life; CS, Cure-allergic-rhinitis Syrup group; YS, Yu-ping-feng San group; GEE, Generalised Estimating Equation test.

T1, baseline at subject recruitment; T2, immediately after intervention; T3, one month after intervention; T4, three months after intervention.

#### Post-hoc comparisons of outcome scores at three post-tests

In Table 7, the results of the pairwise contrast tests following the GEE indicated that the CS group reported significantly greater improvements in:

**Table 7:** Contrast tests of mean score differences on symptom severity, quality of life, and four patterns of body constitution at three post-tests (n = 240)

	**RQLQ-S for SS**		**RQLQ-S for QOL**		**Gentleness**		**Qi-deficiency**		**Yang-deficiency**		**Inherited special**	
	**Slope**	**MD**	**Slope**	**MD**	**Slope**	**MD**	**Slope**	**MD**	**Slope**	**MD**	**Slope**	**MD**
	**(SE)**	**( *P *)**	**(SE)**	**( *P *)**	**(SE)**	**( *P *)**	**(SE)**	**( *P *)**	**(SE)**	**( *P *)**	**(SE)**	**( *P *)**
**T2**												
CS vs. placebo	23.12 (7.59)	-26.13 (0.001)	11.67 (3.96)	-12.81 (0.001)	-3.29 (1.99)	6.18 (0.002)	5.88 (2.56)	-7.87 (0.002)	4.45 (3.08)	-8.53 (0.006)	6.43 (2.45)	-5.22 (0.03)
CS vs. YS	6.61 (7.57)	-5.28 (0.49)	5.05 (3.95)	-4.23 (0.28)	-1.32 (1.99)	3.16 (0.11)	5.74 (2.56)	-2.46 (0.34)	3.97 (3.08)	-5.08 (0.10)	3.24 (2.44)	-1.94 (0.43)
YS vs. placebo	2.33 (7.61)	-20.85 (0.006)	2.05 (3.97)	-8.58 (0.03)	-1.97 (2.01)	3.01 (0.13)	0.14(2.58)	-5.41 (0.04)	0.48 (3.09)	-3.45 (0.27)	3.19 (2.46)	-3.27 (0.18)
**T3**												
CS vs. placebo	29.64 (7.59)	-32.64 (<0.0005)	15.61 (3.96)	-16.76 (<0.0005)	-3.03 (1.99)	5.93 (0.003)	4.49 (2.57)	-6.47 (0.01)	2.35 (3.09)	-6.42 (0.04)	3.79 (2.45)	-2.58 (0.29)
CS vs. YS	12.12 (7.57)	-10.79 (0.15)	6.12 (3.95)	-5.31 (0.18)	-1.00 (1.99)	2.84 (0.15)	6.14 (2.56)	-2.85 (0.26)	4.29 (3.09)	-5.40 (0.08)	2.09 (2.44)	-0.80 (0.74)
YS vs. placebo	2.12 (7.61)	-21.85 (0.004)	2.75 (3.98)	-11.46 (0.004)	-2.03 (2.00)	3.09 (0.12)	-1.65 (2.58)	-3.62 (0.16)	-1.94 (3.10)	-1.03 (0.74)	1.70 (2.46)	-1.78 (0.47)
**T4**												
CS vs. placebo	31.54 (7.59)	-34.54 (<0.0005)	14.71 (3.96)	-15.86 (<0.0005)	0.95 (1.99)	1.95 (0.33)	3.71 (2.56)	-5.70 (0.03)	2.83 (3.05)	-6.90 (0.02)	8.35 (2.44)	-7.14 (0.003)
CS vs. YS	8.80 (7.57)	-7.47 (0.32)	4.93 (3.94)	-4.11 (0.30)	0.57 (1.99)	1.27 (0.52)	4.54 (2.56)	-1.27 (0.62)	3.92 (3.04)	-5.03 (0.10)	4.03 (2.44)	-2.73 (0.26)
YS vs. placebo	2.93 (7.61)	-27.07 (<0.0005)	2.97 (3.97)	-11.74 (0.003)	0.38 (2.00)	0.67 (0.74)	-0.83 (2.57)	-4.44 (0.08)	-1.09 (3.06)	-1.87 (0.54)	4.32 (2.45)	-4.40 (0.07)

RQLQ-S, Rhinoconjunctivitis Quality of Life Questionnaire Standard; SS, Symptom severity; QOL, Quality of life; CS, Cure-allergic-rhinitis Syrup group; YS, Yu-ping-feng San group MD, Mean difference between two groups; SE, Standard error in Generalised Estimating Equation (GEE) test.

T1, baseline at subject recruitment; T2, immediately after intervention; T3, one month after intervention; T4, three months after intervention.

(a) All four patterns of body constitution than the placebo (effect sizes range 0.21 to 0.40), including ‘Qi-deficiency’ and ‘Yang-deficiency’ at all post-tests T2 to T4 (mean difference = -5.70 to -7.87, standard error [SE] = 2.56 to 2.57, *P* = 0.01 to 0.002; mean difference = -6.42 to -8.53, SE = 3.05 to 3.09, *P* = 0.04 to 0.006, respectively); ‘Inherited special’ at two post-tests T2 and T4 (mean difference = -5.22 and -7.14, SE = 2.44 and 2.45, *P* = 0.03 and 0.003, respectively); and ‘Gentleness’ at T2 and T3 (mean difference = 6.18 and 5.93, both SE values = 1.99, *P* = 0.002 and 0.003, respectively).

(b) Symptom severity than the placebo (mean difference = -26.13 to -34.54, all SE values = 7.59, *P* = 0.001 to <0.0005) at T2 to T4 (effect sizes range 0.51 to 0.73); whereas, no significant differences with those in the YS group across the three post-tests.

(c) Quality of life than the placebo (mean difference = -12.81 to –16.76, all SE values = 3.96, *P* = 0.001 to <0.0005) at T2 to T4 (effect sizes range 0.50 to 0.64); whereas, there were no significance differences with those in the YS across the three post-tests.

In addition, the CS group also indicated significantly greater improvements in all of the seven domains of symptom severity (Table 8), including activities, sleep, non-nose/non-eye symptoms, practical problems, nasal symptoms, eye symptoms, and emotional functions, than the placebo group at T2 to T4 (mean difference = -2.04 to -7.95, SE = 0.95 to 2.13, *P* = 0.04 - <0.0005; mean difference = -3.01 to -8.88, SE = 0.95 to 2.13, *P* = 0.008 to <0.0005; mean difference = -3.52 to -7.76, SE = 0.95 - 2.13, *P* = 0.004 to <0.0005, respectively). However, there were consistent but non-significantly greater improvements in all these domains of symptoms in the CS compared to those in the YS group (*P* = 0.99 to 0.11).

**Table 8:** Contrast tests of mean scores of seven domains of symptom severity (in RQLQ-S) between groups at three post-tests (n = 240)

	***Activities***		***Sleep***		***Non-nose/non-eye symptoms***		***Practical problems***		***Nasal symptoms***		***Eye symptoms***		***Emotional***	
	**Slope**	**MD**	**Slope**	**MD**	**Slope**	**MD**	**Slope**	**MD**	**Slope**	**MD**	**Slope**	**MD**	**Slope**	**MD**
	**(SE)**	**( *P *)**	**(SE)**	**( *P *)**	**(SE)**	**( *P *)**	**(SE)**	**( *P *)**	**(SE)**	**( *P *)**	**(SE)**	**( *P *)**	**(SE)**	**( *P *)**
**T2**														
CS vs. placebo	2.02 (0.95)	-2.86 (0.004)	1.78 (0.96)	-2.04 (0.04)	6.39 (2.13)	-7.95 (<0.0005)	3.77 (1.17)	-3.21 (0.006)	3.43 (1.43)	-3.49 (0.02)	2.93 (1.21)	-2.90 (0.02)	2.80 (1.15)	-3.67 (0.001)
CS vs. YS	-0.31 (0.95)	-0.38 (0.69)	0.47 (0.96)	-0.30 (0.76)	2.95 (2.12)	-2.69 (0.20)	1.10 (1.17)	-0.49 (0.68)	0.69 (1.43)	-0.02 (0.99)	0.96 (1.21)	-0.69 (0.57)	0.75 (1.15)	-1.47 (0.20)
YS vs. placebo	2.33 (0.95)	-3.24 (0.002)	1.31 (0.97)	-1.74 (0.07)	3.44 (2.13)	-5.26 (0.01)	2.68 (1.17)	-2.72 (0.02)	2.745 (1.44)	-3.47 (0.02)	1.98 (1.22)	-2.22 (0.07)	2.05 (1.15)	-2.20 (0.06)
**T3**														
CS vs. placebo	2.93 (0.95)	-3.76 (0.001)	2.75 (0.96)	-3.01 (0.002)	7.32 (2.13)	-8.88 (<0.0005)	4.87 (1.17)	-4.31 (<0.0005)	5.44 (1.43)	-5.50 (<0.0005)	3.22 (1.21)	-3.20 (0.008)	3.01 (1.15)	-3.97 (0.001)
CS vs. YS	0.81 (0.95)	-0.74 (0.44)	1.38 (0.96)	-1.21 (0.21)	3.08 (2.12)	-2.82 (0.18)	2.07 (1.17)	-1.46 (0.21)	2.98 (1.43)	-2.30 (0.11)	1.47 (1.21)	-1.20 (0.32)	0.35 (1.15)	-1.06 (0.36)
YS vs. placebo	2.12 (0.95)	-3.03 (0.003)	1.38 (0.97)	-1.80 (0.06)	4.24 (2.13)	-6.06 (0.004)	2.81 (1.17)	-2.85 (0.02)	2.47 (1.44)	-3.20 (0.03)	1.76 (1.22)	-2.00 (0.10)	2.76 (1.15)	-2.91 (0.01)
**T4**														
CS vs. placebo	3.18 (0.95)	-4.01 (<0.0005)	3.47 (0.96)	-3.73 (<0.0005)	6.20 (2.13)	-7.76 (<0.0005)	5.29 (1.17)	-4.73 (<0.0005)	6.70 (1.43)	-6.76 (<0.0005)	3.55 (1.21)	-3.52 (0.004)	3.17 (1.15)	-4.04 (<0.0005)
CS vs. YS	0.25 (0.95)	-0.18 (0.85)	1.29 (0.96)	-1.13 (0.24)	1.61 (2.12)	-1.35 (0.53)	1.37 (1.17)	-0.76 (0.52)	2.91 (1.43)	-2.24 (0.12)	1.18 (1.21)	-0.91 (0.70)	0.19 (1.15)	-0.91 (0.43)
YS vs. placebo	2.93 (0.95)	-3.83 (0.001)	2.18 (0.97)	-2.60 (0.007)	4.59 (2.13)	-6.41 (0.003)	3.92 (1.17)	-3.97 (0.001)	3.79 (1.44)	-4.52 (0.002)	2.36 (1.22)	-2.61 (0.03)	2.97 (1.15)	-3.12 (0.007)

CS, Cure-allergic-rhinitis Syrup group; YS, Yu-ping-feng San group; RQLQ-S, Rhinoconjunctivitis Quality of Life Questionnaire Standard; MD, Mean difference between two groups; SE, Standard error in GEE test.

As shown in Tables 7 and 8, the YS group reported significantly greater improvements in the overall and four domains of symptom severity at T2 to T4 (effect sizes ranged 0.22 to 0.41) than the placebo group, including total score (mean difference = -20.85 to -27.07, all SEs = 7.61, *P* = 0.006 to <0.0005), activities (mean difference = -3.03 to -3.83, all SEs = 0.95, *P* = 0.003 to 0.001), non-nose/non-eye symptoms (mean difference = -5.26 to -6.41, all SEs = 2.13, *P* = 0.01 to 0.003), practical problems (mean difference = -2.72 to -3.97, all SEs = 1.17, *P* = 0.02 to 0.001), and nasal (mean difference = -3.20 to -4.52, all SEs = 1.44, *P* = 0.03 to 0.002). The YS group also showed significantly greater improvements in the domains of emotional function at T3 and T4 (mean difference = -2.91 and -3.12, both SEs = 1.15, *P* = 0.01 and 0.007), and sleep (mean difference = -2.60, SE = 0.97, *P* = 0.007) and eye symptoms (mean difference = -2.61, SE = 1.22, *P* = 0.03) at T4, when compared to the placebo group (effect sizes ranged 0.29 to 0.37).

For the current usage of Western medicine, a total of 62 participants (25.8%) received one to a few types of Western medicine for treatment of AR according to doctors’ prescription or over-the-counter medications (n = 30 in CS; n = 15 in YS; and n = 17 in placebo) before this trial. However, there was a significant reduction of such medication use in both the CS (n = 1) and YS (n = 3) groups over the 3-month follow-up; whereas, there were only slight changes in medication use among the placebo controls (i.e., n = 14 over follow-up).

## Discussion

The findings of this study are encouraging to support the positive effects of Chinese herbal medicinal formula Cure-Allergic Rhinitis Syrup [治敏膏] (CS) when treating young adults (nursing students) with AR. The CS was found to significantly improve the nursing students’ symptom severity, body constitution, and quality of life over the 3-month follow-up. The findings demonstrated that an application of herbal medicine with ‘Hot’ and ‘Qi’ (and ‘Yang’) rectifying nature, that was the CS tested in this trial, could strengthen functions of the ‘Lung’ (respiratory organ) and ‘Spleen’ (digestive organ), as well as the ‘Kidney’ (excretory organ), thus alleviating the symptoms of AR [27,28]. Over the 3-month follow-up, the ‘Qi-deficiency’, ‘Yang-deficiency’, and ‘Inherit special’ (i.e., the inherited entities that guide one’s body development and formation of special characters such as allergic reactions) patterns of body constitution were much improved, while the ‘Gentleness’ (healthy) body constitution had increasingly strengthened, when compared to the placebo group. Overall, most of these significant effects (‘Qi-deficiency’, ‘Yang-deficiency’, and ‘Inherit special’) in the CS group could also be obtained much earlier and last longer than those in the YS group. As supported by the theories of CM, the CS could facilitate the balance between ‘Yin’ and ‘Yang’ by regulating or rectifying ‘Qi’ flowing inside the body and thus relieve the deficiency of ‘Yang’, which kept motivating the functions of the internal organs and produce energy in the body parts [45].

The participants in this trial, similar to most people with AR, presented with subnormal regulation of thermal and/or body defence conditions, which was innate to human beings [46]. They might require for the herbal medicine (i.e., the CS), or other regulatory forces, to strengthen or enhance their ‘Qi’ to drive the whole body (body organs and systems) to keep warm, maintain a good immune defence, and promote the internal regulatory mechanisms [47]. The majority of AR sufferers, like the nursing students in this trial, have syndromes (‘*Zheng*’) of ‘Qi-deficiency’ and/or ‘Yang-deficiency’ with ‘Cold’ or ‘flu’ like symptoms such as running nose and nasal congestion. These could be treated by using herbal medicines with ‘Hot’ or ‘Warm’ in nature, which were particularly useful to regulate the malfunctions of the respiratory (‘Lung’) and digestive (‘Spleen’) systems, and even excretory (‘Kidney’) organs [45,46].

The findings also provided evidence on the significance of prescription of Chinese herbal medicines based on assessment results of syndrome (‘*Zheng*’) and body constitution, which reflected the structure, metabolism, and functioning of the main body organs that contributed to determining one’s susceptibility to pathogenic factors [14,15]. All participants in this trial were assessed by a CM practitioner to confirm their ‘Qi-deficiency’ and/or ‘Yang-deficiency’ before being included in this study and provided them with the CS or YS. Accurate prescription of medicine based on accurate ‘*Zheng*’ differentiation can assure or enhance its therapeutic or curative effects to an illness. On the other hand, neglecting the syndrome differentiation or misinterpreting the body constitution may result in inappropriate treatment prescription, thus causing unsatisfactory or even harmful and adverse effects to treatment recipients. Only four patients (1.6%) in this trial reported adverse effects, which was much lower than other clinical trials of herbal medicinal treatment of AR without ‘*Zheng*’ identification, for example, about 21% in Xue et al.’s [19] study.

In addition, this trial reported a low attrition rate (n = 9, 3.6%), in which only one participant in the CS group was unable to complete the 4-week treatment and was lost to follow-up. As patterns of body constitution signify the reactions of the body to specific pathogen(s), the participants (nursing students) with AR who were unable to keep warm and satisfactory immune defences had developed hypersensitive reactions to those allergens, thus resulting in main symptoms of AR [46,48]. It is noteworthy that two strategies adopted in this trial might facilitate the participants’ completion of their treatments, including regular consultation and assessment of their health condition during the treatment period and the use of medication in syrup format which was considered more user-friendly, with a better taste and more accurate dosage than conventional administration of herbal medicine. Therefore, the 4-week CS treatment was found to be more consistent and have substantive therapeutic effects on patient outcomes than other clinical trials of herbal medicinal treatment of AR [15,17-21]. Nevertheless, future research in a large-sized, representative sample with diverse socio-demographic and illness-related characteristics (e.g., wider age ranges, living environments, and family medical history), and a longer-term follow-up (e.g., one year), is recommended to examine a more consistent and conclusive curative effect of the CS before its application for usual treatment.

Yu-ping-feng San [玉屏風散] (YS), which contained six Chinese herbs (plus sugar and wheat flour) commonly used for treatment of AR in recent CM studies [22,23], was adopted to be the alternative medicinal treatment in this trial. The results indicated its small to moderate effects on improving the participants in their symptom severity, quality of life, and one to two patterns of body constitution over the 3-month follow-up. When compared to the CS group, the participants in the YS group showed less prominent and substantive effects on the main treatment outcomes, particularly their body constitution. The YS has been used in recent clinical trials of AR to strengthen the ‘Qi’, allaying nasal symptoms and protect the ‘Lung’ and ‘Spleen’ from pathological invasions, but not including the ‘Kidney’ organ [32-34,49,50]. Therefore, the YS might not be able to fully reinforce and rectify the ‘Qi-Yang’ in the three target organs, thus being inadequate to minimise the symptoms of AR or cure AR in the long term, as the CS did.

### Limitations and future research

A few limitations of this study were found. First, the sample that was a group of nursing students voluntarily participated (i.e., <20% of the eligible subjects) and recruited from only one of the three universities in Hong Kong. The findings, although promising, might not be generalizable to other Chinese young adults or different age groups with AR. The participants might also be highly motivated for treatment due to their nursing background. Second, risk factors influencing symptoms of AR could not be similar between different years of nursing study, for example, there were relatively much longer periods of clinical placement among those senior students who would therefore be more frequently exposed to disinfectants and other allergens in hospitals and other clinical units. Third, this study adopted a 3-month follow-up period. Only about 12-16% of them (n = 10-13) in the CS group indicated an absence of Qi-deficiency, Yang-deficiency, and Inherited special BC at the post-tests T2- T4. AR is an enduring and distressing chronic illness, the substantive effect of herbal medicine used to cure and prevent reoccurrence of the illness should be examined with a longer term follow-up (e.g., >1 year).

However, the ideal of a double-blind, randomised controlled trial could be achieved due to the complete concealment of the participants and research team to the treatment assignment and very similar study procedure and spicy and syrup form of medications. However, it is recommended to explore the perceived benefits and limitations of the treatment experienced from the perspective of the participants in order to better understand the treatment procedure, progress, and strengths of the treatment offered, as well as its room for improvement.

Future research can include other outcome measures used in Western medicine such as the blood test for IgE and skin sensitivity tests for allergens to confirm and/or compare the clinical efficacy of the Chinese herbal medicine used across studies. Furthermore, research to compare the relative clinical efficacy between the CS and other approaches to Chinese and/or Western medicine or complementary and alternative therapies, such as dietary and massage therapy, on a wide variety of patient outcomes can be considered.

## Conclusions

This double-blind randomised controlled trial demonstrated that the herbal medicinal formula CS could be effective in Chinese young adults (nursing students) with AR and ‘Yang- and/or Qi-deficiency’ patterns of body constitution (or ‘*Zheng*’). The herbal medicines in syrup form were found to be user-friendly and useful to significantly reduce the participants’ symptoms of AR and improve their body constitution and quality of life over three months of follow-up. The findings also suggest that the syndrome differentiation facilitated the accurate treatment prescription, making the herbal medicine used more appropriate to the patients, thus resulting in significantly better therapeutic or curative effects. Further research is recommended to test the clinical efficacy of the CS in diverse patient populations and a long-term follow-up to confirm its curative function in the treatment of allergic rhinitis.

## Abbreviations

AR: Allergic rhinitis; CCMQ: Constitution in Chinese Medicine Questionnaire; CM: Chinese medicine; CS: Cure-allergic-rhinitis Syrup; GEE: Generalized estimating equation test; RQLQ-S: Rhinoconjunctivitis quality of life questionnaire standard; YS: Yu-ping-feng San.

## Competing interests

The authors declare that they have no competing interests.

## Authors’ contributions

RYPC contributed to the literature search and review, study design and implementation, data collection, data analysis, and preparation of the manuscript; WTC contributed to the literature review, study design, data analysis, and editing and finalising the manuscript. Both authors read and approved the final manuscript.

## Authors’ information

Rose YP Chan, MSc, BSc, RN, RCMP is an experienced registered Chinese medicine practitioner and registered nurse in Hong Kong, and is studying for a PhD on treatments of allergic rhinitis in the School of Nursing, Faculty of Health and Social Sciences at the Hong Kong Polytechnic University (Hung Hom, Kowloon, Hong Kong SAR, China).

Wai Tong Chien, PhD, MPhil, BN, RMN, is a professor and mental health research group leader in the School of Nursing, Faculty of Health and Social Sciences at The Hong Kong Polytechnic University (Hung Hom, Kowloon, Hong Kong SAR, China). Prof. Chien is also experienced in randomised controlled trials with extensive publications about treatments or interventions for a wide variety of acute and chronic diseases such as allergic rhinitis, depression, psychotic disorders, and dementia.

## References

[B1] Rondón C Fernandez J Canto G Blanca M Local allergic rhinitis: concept, clinical manifestations, and diagnostic approach J Investig Allergol Clin Immunol 2010 20 364 371 20945601

[B2] Guo R Pittler MH Ernst E Herbal medicines for the treatment of allergic rhinitis: a systematic review Ann Allergy Asthma Immunol 2007 99 483 495 18219828 10.1016/S1081-1206(10)60375-4

[B3] Shea KM Truckner RT Weber RW Peden DB Climate change and allergic disease J Allergy Clin Immunol 2008 122 443 453 18774380 10.1016/j.jaci.2008.06.032

[B4] van Cauwenberge P Bachert C Passalacqua G Bousquet J Canonica GW Durham SR Fokkens WJ Howarth PH Lund V Malling HJ Mygind N Passali D Scadding GK Wang DY Consensus statement on the treatment of allergic rhinitis Allergy 2000 55 116 134 10726726 10.1034/j.1398-9995.2000.00526.x

[B5] Spector SL Overview of co-morbid associations of allergic rhinitis J Allergy Clin Immunol 1997 99 S773 S780 9042070 10.1016/s0091-6749(97)70126-x

[B6] Meltzer EO Blaiss MS Derebery MJ Mahr TA Gordon BR Sheth KK Simmons AL Wingertzahn MA Boyle JM Burden of allergic rhinitis: results from the pediatric allergies in America survey J Allergy Clin Immunol 2009 124 S43 S70 19592081 10.1016/j.jaci.2009.05.013

[B7] Zaslawki C Clinical reasoning in traditional Chinese medicine: implications for clinical research Clin Acupunct Oriental Med 2003 4 94 101

[B8] Jiang WY Therapeutic wisdom in traditional Chinese medicine: a perspective from modern science Trends Pharmacol Sci 2005 26 558 563 16185775 10.1016/j.tips.2005.09.006

[B9] Zhou ST Kuang DY Clinical experience of treatment based on constitution differentiation by Professor Kuang Diao-Yuan (Two)-Clinical Gynecology Chin Arch Tradit Chin Med 2012 30 2603 2608

[B10] Jiang F Relationship between allergic rhinitis and Chinese medical constitution Chin J Tradit Chin Med Pharm 2008 23 140 142

[B11] Yang S Chen H Lin Y Chen Y The exploration of disease pattern, Zheng, for differentiation of allergic rhinitis in traditional Chinese medicine practice Evid Based Complement Alternat Med 2012 2012 521780 22899954 10.1155/2012/521780 PMC3414236

[B12] Wang RX Zhang CH Ning YH Discussion and treatment of Biqiu based on the weakness of the viscera Shan Dong Zhong Yi Za Zhi 2009 28 80 81

[B13] Kung YY Chen YC Hwang SJ Chen TJ Chen FP The prescriptions frequencies and patterns of Chinese herbal medicine for allergic rhinitis in Taiwan Allergy 2006 61 1316 1318 17002708 10.1111/j.1398-9995.2006.01152.x

[B14] Liang KL Jiang RS Lee CL Chiang PJ Lin JS Su YC Traditional Chinese medicine Zheng identification provides a novel stratification approach in patients with allergic rhinitis Evid Based Complement Alternat Med 2012 2012 480715 22745648 10.1155/2012/480715 PMC3383108

[B15] Chui SH Shek SL Fong MY Szeto YT Chan K A panel study to evaluate quality of life assessments in patients suffering from allergic rhinitis after treatment with a Chinese herbal nasal drop Phytother Res 2010 24 609 613 20014162 10.1002/ptr.3058

[B16] Lenon GB Li CG Da Costa C Thien FCK Shen Y Xue CCL Lack of efficacy of a herbal preparation (RCM-102) for seasonal allergic rhinitis: a double blind, randomized, placebo-controlled trial Asia Pac Allergy 2012 2 187 194 22872821 10.5415/apallergy.2012.2.3.187 PMC3406298

[B17] Zhao Y Woo KS Ma KH van Hansselt CA Wong KC Cheng KF Lam CW Leung PC Treatment of perennial allergic rhinitis using Shi-Bi-Lin, a Chinese herbal formula J Ethnopharmacol 2009 122 100 105 19118617 10.1016/j.jep.2008.12.005

[B18] Hu G Walls RS Bass D Bullock R Grayson D Jones M Gebski V The Chinese herbal formulation Biminne in management of perennial allergic rhinitis: a randomized, double-blind, placebo-controlled, 12-week clinical trial Ann Allerg Asthma Im 2002 88 478 487 10.1016/s1081-1206(10)62386-1 12027069

[B19] Xue CCL Thien FC Zhang JJ Da Costa C Li CG Treatment for seasonal allergic rhinitis by Chinese herbal medicine: a randomized placebo controlled trial Altern Ther Health Med 2003 9 80 87 14526714

[B20] Yang SH Yu CL Antiinflammatory effects of Bu-zhong-yi-qi-tang in patients with perennial allergic rhinitis J Ethnopharmacol 2008 115 104 109 17980528 10.1016/j.jep.2007.09.011

[B21] Yang SH Chia LY Chen YL Chiao SL Chen ML Traditional Chinese medicine, Xin-yi-san, reduces nasal symptoms of patients with perennial allergic rhinitis by its diverse immunomodulatory effects Int Immunopharmacol 2010 10 951 958 20546945 10.1016/j.intimp.2010.05.008

[B22] Wen J Zhu JM Li J Yuan GM Xiang FJ Experimental study of Yupingfen Granule on allergic rhinitis in rat and guinea pig Chin Tradit Patent Med 2011 33 934 937

[B23] Lei XQ Clinical analysis of treating allergic rhinitis with Yiqi Jianbi decoction Chin J Chin Med 2011 3 55 56

[B24] Yan XL Allergic rhinitis treated with modified Yu Ping Feng San in 115 cases J Beijing Univ Tradit Chin Med 2011 34 358 360

[B25] Wang P Tentative discussion on difference betwe*en XIAOJIANZH*ONG and LIZHONG decoctions J Fujian College Tradit Chin Med 2003 13 46 48

[B26] Shyue SK The study of the relationship between gene and protein e*xpression and* the Chinese medical constitutional types in the patients of allergic rhinitis-role of cytokines Yearbook of Chinese Medicine and Pharmacy 2008 26 205 236

[B27] Fu XD The ten methods used by Dr Chan Guo-feng for treating allergic rhinitis J Tradit Chin Med 2010 30 206 210 21053628 10.1016/s0254-6272(10)60042-8

[B28] Chen FP Chen FJ Jong MS Tsai HL Wang JR Hwang SJ Modern use of Chinese herbal formulae from Shang-Han Lun Chin Med J 2009 122 1889 1894 19781366

[B29] Schulz KF Altman DG Moher D for the CONSORT Group CONSORT 2010 Statement: updated guidelines for reporting parallel group randomised trials BMJ 2010 340 c332 20332509 10.1136/bmj.c332 PMC2844940

[B30] Jung JW Kang HR Ji GE Park MS Song WJ Kim MH Kwon JW Kim TW Park HW Cho SH Min KU Therapeutic effects of Fermented Red Ginseng in allergic rhinitis: a randomized, double-blind, placebo-controlled study Allergy Asthma Immunol Res 2011 3 103 110 21461249 10.4168/aair.2011.3.2.103 PMC3062788

[B31] Freedman KB Back S Bernstein J Sample size and statistical power of randomized, controlled trials in orthopaedics J Bone Joint Surg 2001 83-B 397 402 10.1302/0301-620x.83b3.10582 11341427

[B32] Cunningham JB McCrum-Gardner E Power, effect and sample size using GPower: practical issues for researchers and members of research ethics committees Evid Based Midwifery 2007 5 132 136

[B33] Allergic Rhinitis and Its Impacts on Asthma Management of Allergic Rhinitis and its Impact on Asthma-Pocket Guide 2007 http://www.whiar.org/docs/ARIA_PG_08_View_WM.pdf, http://www.whiar.org/Documents&Resources.php#1

[B34] Tsai PF Chen CL Yang SH The correlation between the three traditional Chinese medicine patterns of allergic rhinitis and age J Chin Med 2010 21 43 52

[B35] Wang Q Zhu YB Xue HS Li S Primary compiling of constitution in Chinese Medicine Questionnaire Chin J Clin Rehabilit 2006 10 12 14

[B36] Hu H Shatanati M Biken A Adali B Deng N Wang ZZ Li J Chwn YP Liu PZ Epidemiological studies of TCM constitutions of Xinjiang Kazakh people with allergic rhinitis J Xinjiang Medical Univ 2013 36 681 684

[B37] Wang Q Zhu YB Classification and Determination of Constitution in TCM 2009 Bejing: Zhongguo Zhongyiyao Chubanshe

[B38] Wong W Lam CLK Wong VT Yang ZM Ziea ETC Kwan AKL Validation of constitution in Chinese Medicine Questionnaire: does the traditional Chinese medicine concept of body constitution exist? Evid Based Complement Alternat Med 2013 2013 481491 23710222 10.1155/2013/481491 PMC3655622

[B39] Juniper EF Guyatt GH Development and testing of a new measure of health status for clinical trials in rhinoconjunctivitis Clin Exp Allergy 1991 21 77 83 2021881 10.1111/j.1365-2222.1991.tb00807.x

[B40] Juniper EF Guyatt GH Griffith LE Ferrie PJ Interpretation of rhinoconjunctivities quality of life questionnaire data J Allergy Clin Immunol 1996 98 843 845 8876562 10.1016/s0091-6749(96)70135-5

[B41] Bousquet J Neukirch F Bousquet PJ Gehano P Klossek JM Gal ML Allaf B Severity and impairment of allergic rhinitis in patients consulting in primary care J Allergy Clin Immunol 2005 117 158 162 16387600 10.1016/j.jaci.2005.09.047

[B42] Bousquet PJ Combescure C Neukirch F Klossek JM Mechin H Daures JP Bousquet J Visual analog scales can assess the severity of rhinitis graded according to ARIA guidelines Allergy 2007 62 367 372 17362246 10.1111/j.1398-9995.2006.01276.x

[B43] Juniper EF Thompson AK Ferrie PJ Roberts JN Validation of the standardized version of the Rhinoconjunctivities Quality of Life Questionniare J Allergy Clin Immunol 1999 104 364 369 10452758 10.1016/s0091-6749(99)70380-5

[B44] Ballinger GA Using generalized estimating equations for longitudinal data analysis Organ Res Meth 2004 7 127 150

[B45] Chan RYP Chien WT Concepts of body constitution, health and sub-health from traditional Chinese medicine perspective World J Transl Med 2013 2 56 66

[B46] Graudenz GS Landgraf RG Jancar S Tribess A Fonseca SG Fae KC Kalil J The role of allergic rhinitis in nasal responses to sudden temperature changes J Allergy Clin Immunol 2006 118 1126 1132 17088139 10.1016/j.jaci.2006.07.005

[B47] Maciocia G The Foundations of Chinese Medicine a Comprehensive Text for Acupuncturists and Herbalists 2005 2 Nanjing: Elsevier Churchill Livingstone 41 61

[B48] Maciocia G Allergic Rhinitis The Practice of Chinese Medicine the Treatment of diseases with Acupuncture and Chinese Herbs 2008 2 Toronto: Churchill Livingstone Elsevier 177 199

[B49] Makino T Sakaki SY Ito Y Kano Y Pharmacological properties of traditional Medicine (XXX): effects of Gyokuheifusan (玉屏封散) on murine antigen-specific antibody production Biol Pharm Bull 2005 28 110 113 15635173 10.1248/bpb.28.110

[B50] Li M Bai H Li SL Therapeutic effect of herbal medicines with the effects of Qi-boosting and exterior-securing on allergic rhinitis Chin Otorhinolaryngol J Integ Med 2007 15 23 25

